# ‘The mirror of the soul?’ Inferring sadness in the eyes

**DOI:** 10.1038/s41598-024-68178-0

**Published:** 2024-08-29

**Authors:** Jonas Moosavi, Annika Resch, Alexander N. Sokolov, Andreas J. Fallgatter, Marina A. Pavlova

**Affiliations:** 1 Social Neuroscience Unit, Department of Psychiatry and Psychotherapy, Tübingen Center for Mental Health (TüCMH), Medical School and University Hospital, Eberhard Karls University of Tübingen, Calwerstr. 14, 72076 Tübingen, Germany; 2 German Center for Mental Health (DZPG), Partner Site Tübingen, Germany

**Keywords:** Basic emotions, Sadness, Reading emotions in the eyes, Face covering, Poser gender, COVID-19, Neuroscience, Social neuroscience

## Abstract

The eyes are widely regarded as the mirror of the soul, providing reliable nonverbal information about drives, feelings, and intentions of others. However, it is unclear how accurate emotion recognition is when only the eyes are visible and whether inferring of emotions is altered across healthy adulthood. To fill this gap, the present piece of research was directed at comparing the ability to infer basic emotions in two groups of typically developing females that differed in age. We set a focus on females seeking group homogeneity. In a face-to-face study, in a two-alternative forced choice paradigm (2AFC), participants had to indicate emotions for faces covered by masks. The outcome reveals that although the recognition pattern is similar in both groups, inferring sadness in the eyes substantially improves with age. Inference of sadness is not only more accurate and less variable in older participants, but also positively correlates with age from early through mid-adulthood. Moreover, reading sadness (and anger) is more challenging in the eyes of male posers. A possible impact of poser gender and cultural background, both in expressing and inferring sadness in the eyes, is highlighted.

## Introduction

The eyes are believed to be the windows to the soul, delivering nonverbal, reliable information about emotional states and personal traits of a social counterpart. In other words, one can understand a person’s emotions, intentions, drives and desires by simply looking into his or her eyes. This view had been recently questioned in the context of the COVID-19 pandemic, leading to mandatory mask wearing that leaves primarily the information from the eyes available for social communication and interaction. Indeed, how accurate is emotion recognition when only the eyes are visible? Recent studies repeatedly report that reading emotions behind a mask remains efficient for some basic expressions such as happiness, but inferring other emotions such as disgust and sadness may be heavily affected^1–16^. Yet, cultural differences in emotional expressions as well as some methodological issues, such as the limitations of online research, may substantially contribute to inconsistency and low replicability of some findings, for instance, for anger recognition^17–19^.

Pre-pandemic abilities for reading language of the eyes, as assessed by the Reading the Mind in the Eyes Test (RMET^20^; with visual input comparable with masked faces; for review see^21^), are shown to improve during the pandemic in female adults, as well as male and female adolescents^22^. In line with this, experience in viewing people wearing face masks leads to better emotion recognition in Brazilian and Swiss preschoolers aged 4 to 6 years^23^. This suggests a kind of implicit or passive learning. On the other hand, a comparison of emotion recognition in two independent groups of participants in May 2020 and July 2021 suggests that even after more than a year of the COVID-19 pandemic, masks remain a burden for recognizing emotions^5^. At the same time, individuals who self-reportedly are more practiced in watching and regularly interacting with people wearing masks are more effective and confident in recognizing facial expressions^5^. On the same wavelength, the face-specific event-related potentials (ERPs, N170 and P2) are reported to be affected by mask wearing, and the impact is more pronounced in individuals with less experience with masked faces^24^. Therefore, as expected^17^, the brain appears to adapt to some constraints in decoding social input.

The question arises, whether inferring facial affect from the eyes improves with life experience. By contrast with other cognitive abilities such as working memory, nonverbal social cognition is believed to remain relatively intact with age^25,26^. Recent research indicates a lack of deterioration or even an improvement in reading language of the eyes as assessed by the RMET in healthy aging^27,28^. In accordance with this, as compared to younger people (aged 18–33 years), older adults (aged 51–83 years) exhibit equal accuracy and difficulties in inferring happiness, sadness, and disgust in masked faces^29^. The effect of masks on emotion recognition (anger, fear, contempt, and sadness) in dynamic facial expressions does not differ between younger (18–30 years) and older (65–85 years) adults^30^.

It is largely unknown whether inferring emotions from the eyes is altered across more comparable intervals of the lifespan, namely, from young adulthood to middle age. To fill this gap, the present piece of research was directed at the assessment of inferring basic emotions in two groups of typically developing (TD) individuals that differ in age. In search of group homogeneity, a focus has been set on female participants, as gender differences are reported in recognition of emotions in masked faces^19,31,32^.

## Materials and methods

### Participants

Twenty-five female TD participants (Group 1) were recruited in February–March 2023 to examine gender differences in inferring emotions in masked faces^19^. Thirty TD female participants (Group 2) were engaged as controls for our earlier study in females with major depressive disorder (MDD) from April 2023 until February 2024^33^. None of them had head injuries, a history of mental disorders, or regular medication. They were recruited from the local community. Participants of Group 1 were aged 22.28 ± 3.34 years (mean±standard deviation, SD; median, Mdn, 22 years, 95% confidence interval, CI [21.74; 22.82]), and those of Group 2 were aged 36.80 ± 13.12 years (Mdn, 33 years, 95% CI [31.90; 41.70]), with a significant age difference between the groups (Mann–Whitney test, *U* = 106.0, *p* < 0.001, two-tailed). German as a native language was used as one of the inclusion criteria. As cultural background affects emotion recognition in masked faces^34^, one of our inclusion criteria was growing up and permanently living in Germany. All observers had normal or corrected-to-normal vision. They were tested individually and were naïve as to the purpose of the study. The study was conducted in line with the Declaration of Helsinki and approved by the local Ethics Committee at the Medical School, Eberhard Karls University of Tübingen. Informed written consent was obtained from all participants. Participation was voluntary and the data was processed anonymously.

### Emotions in masked faces (EMF) task

**Figure 1:**
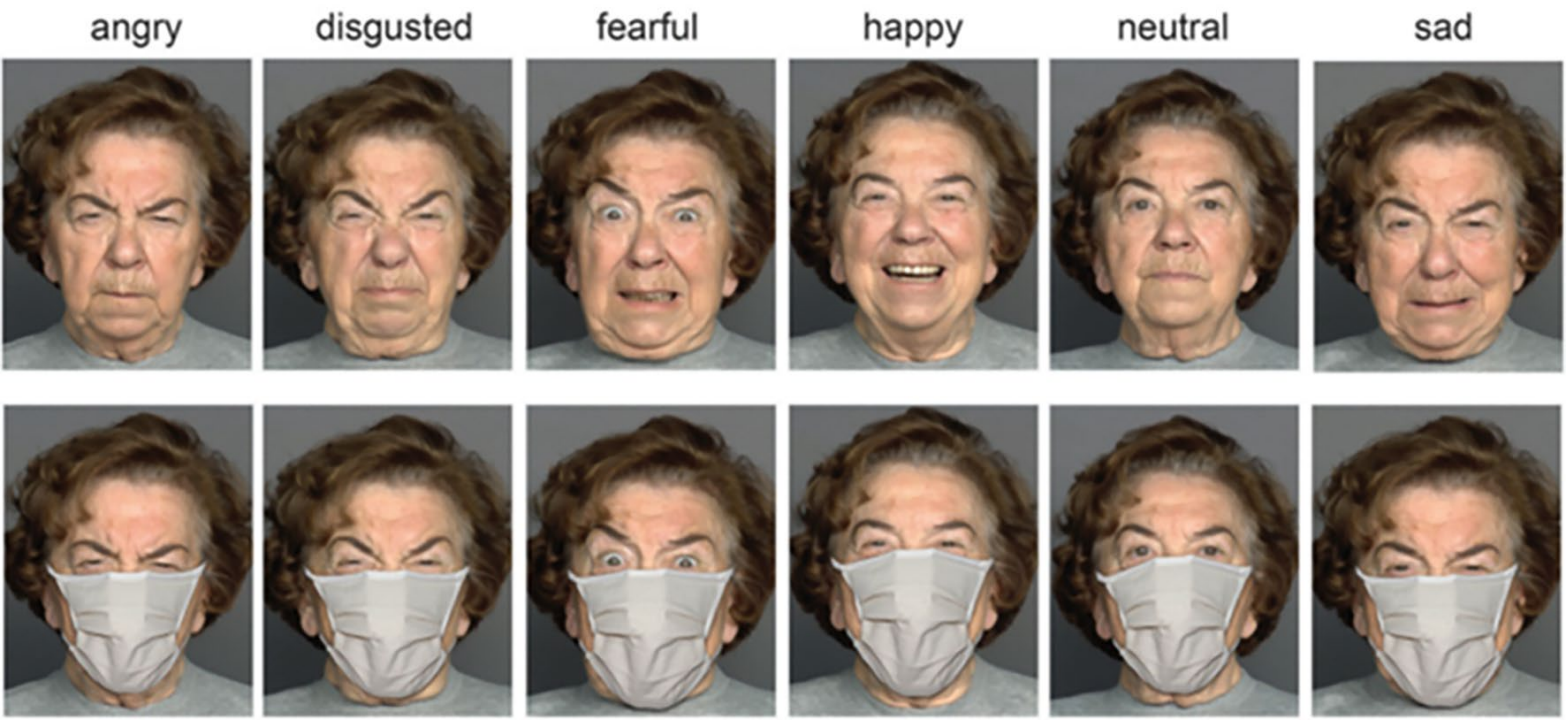
A female poser expressing six basic emotions. Faces are shown under full-face (top) and covered-by-mask conditions (bottom row). From Carbon^1^, the Creative Commons Attribution [CC BY] license. These images are presented for illustrative purposes only, and have not been used as experimental material in the present study.

The stimuli and task are described in detail elsewhere^19^. In brief, photographs of six (three female and three male) Caucasians from three distinct age groups (young, middle, and older age), were taken from the Max Planck Institute FACES database^35^ with permission (see Fig. 1). Each depicted person displayed six emotional states (anger, disgust, fear, happiness, sadness, and neutral expressions; a surgical face mask was applied to all faces). This resulted in 36 images and a total of 108 trials (6 emotions × 2 genders × 3 age groups × 3 runs). Two alternative (correct/incorrect) responses for each trial were used, which were chosen based on the emotion confusion data^1,5^: *angry—disgusted, fearful—sad,* and *neutral—happy.* On each trial, in a two-alternative forced choice paradigm (2AFC), participants had to indicate the correct emotion. For avoiding possible implicit learning effects, only masked faces were used. Participants were administered a computer version of the EMF task by using Presentation software (Neurobehavioral Systems, Inc., Albany, CA, USA). The stimuli were presented in a pseudo-randomized order, one at a time for 2 s, in three runs separated by short breaks. Upon image offset, a correct and incorrect response appeared on the right and left sides of a screen. Participants were asked to respond as accurately as possible. Participants were carefully instructed and their understanding had been proven with pre-testing (about ten trials) performed under supervision of an examiner. No immediate feedback was provided. The testing lasted for about 15–20 min per person.

### Data processing and analysis

All data sets were checked for normality of distribution by means of the Shapiro–Wilk test. For non-normally distributed data, additionally to means and SDs, Mdns and 95% CIs were reported. For normally distributed data, a linear Pearson correlation was calculated, whereas for non-normally distributed data, a non-linear Spearman correlation was used. Inferential statistics was performed by analyses of variance (ANOVAs) and pairwise comparisons with the software package JMP (version 16; SAS Institute, Cary, NC, USA). Non-parametric statistics was performed using MATLAB (version 2023a; MathWorks, Inc., Natick, MA, USA).

## Results

### Recognition accuracy

Individual correct response rates for each emotion of the EMF task were submitted to a two-way mixed-model ANOVA with the within-subject factor Emotion (angry, happy, neutral, sad, fearful, disgusted) and between-subject factor Group (Group 1/Group 2). As expected, a main effect of Emotion was highly significant (*F*(5,316) = 62.74, *p* < 0.001, effect size, *eta-squared η*^2^ = 0.54). A main effect of Group (*F*(1,316) = 0.004, *p* = 0.951, n.s.) as well as a Group by Emotion interaction (*F*(5,316) = 1.78, *p* = 0.116, n.s.) were not significant.

Both Groups 1 and 2 exhibited a similar uneven pattern of emotion recognition: inferring facial affect in the eyes was rather accurate for fear, neutral expressions, and happiness, whereas it was less precise for disgust and sadness. This outcome indicates the replicability of our earlier findings^19^.

**Figure 2:**
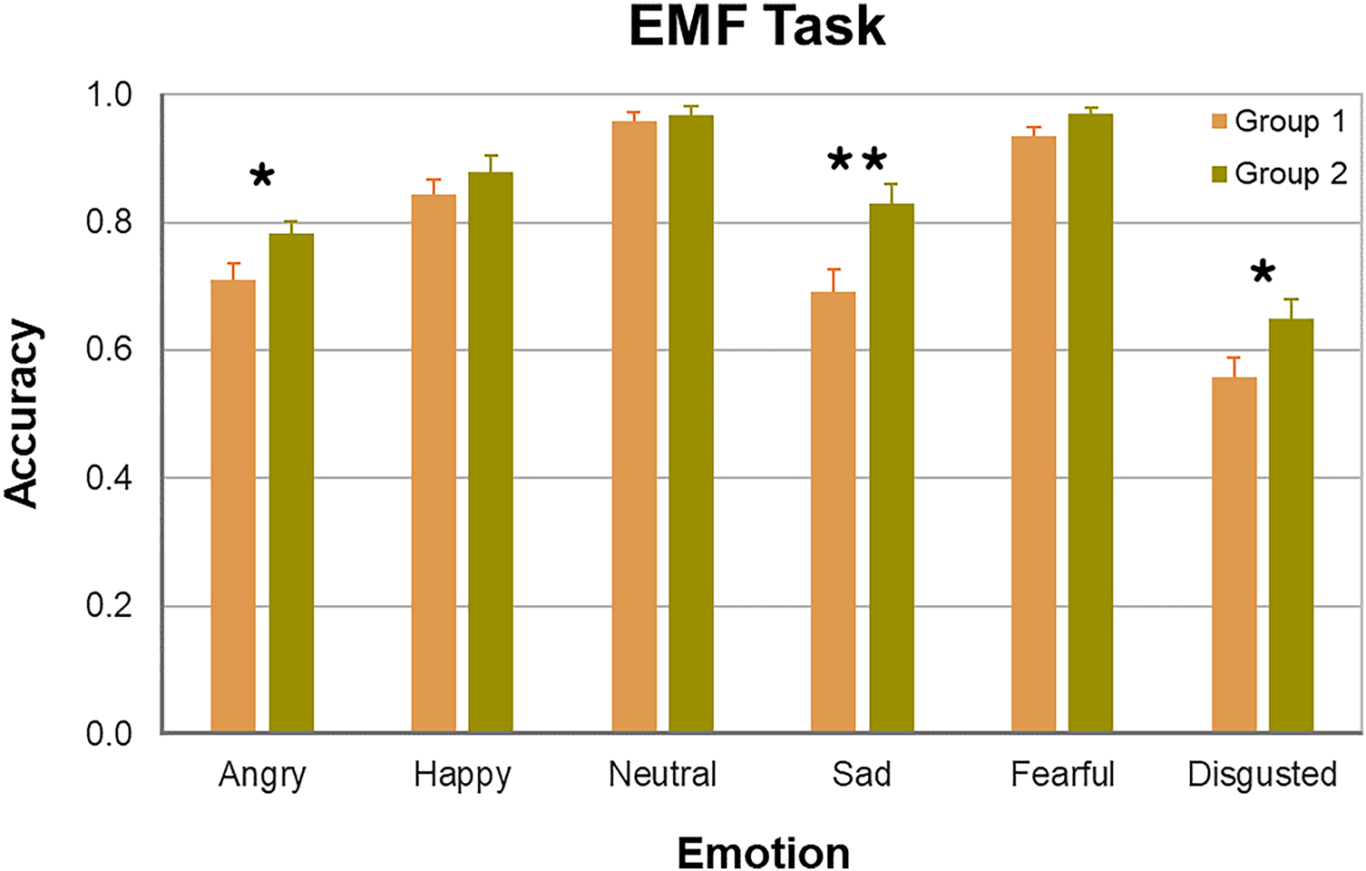
Recognition accuracy of facial emotions on the EMF task for Group 1 (apricot, from Pavlova et al.^19^) and Group 2 (olive green; from Moosavi et al.^33^). Vertical bars represent ± SEM. Double asterisk indicates a significant difference (*p* < 0.05), single asterisks a tendency (0.05 < *p* < 0.1).

As seen in Fig. 2, pairwise comparisons revealed a significant difference between the groups in inferring sadness (Group 1, 0.691 ± 0.193; Group 2, 0.830 ± 0.162, Mdn, 0.889, 95% CI [0.769; 0.890]; *U* = 201, *p* = 0.018, here and further false discovery rate [FDR] corrected for multiple comparisons [*p* = 0.003, uncorrected] and two-tailed, effect size, Cohen’s* d* = 0.86). The difference in inferring anger (Group 1, 0.709 ± 0.146; Group 2, 0.783 ± 0.104, Mdn, 0.833, 95% CI [0.745; 0.822]; *U* = 257, *p* = 0.047, uncorrected, *d* = 0.56) and disgust (Group 1, 0.558 ± 0.165; Group 2, 0.650 ± 0.159; *t*(53) = 2.09, *p* = 0.041, uncorrected, *d* = 0.57) was also significant, but did not survive corrections for multiple comparisons and only tended to reach significance (anger, *p* = 0.094; disgust, *p* = 0.094). For other emotions, no significant differences in recognition accuracy were revealed between Group 1 and Group 2 (Table S1, Supplementary Material). This finding suggests that in inferring subtle emotions such as sadness in the eyes, experience obtained with age may be of substantial advantage.

### Link of EMF task with age

**Figure 3:**
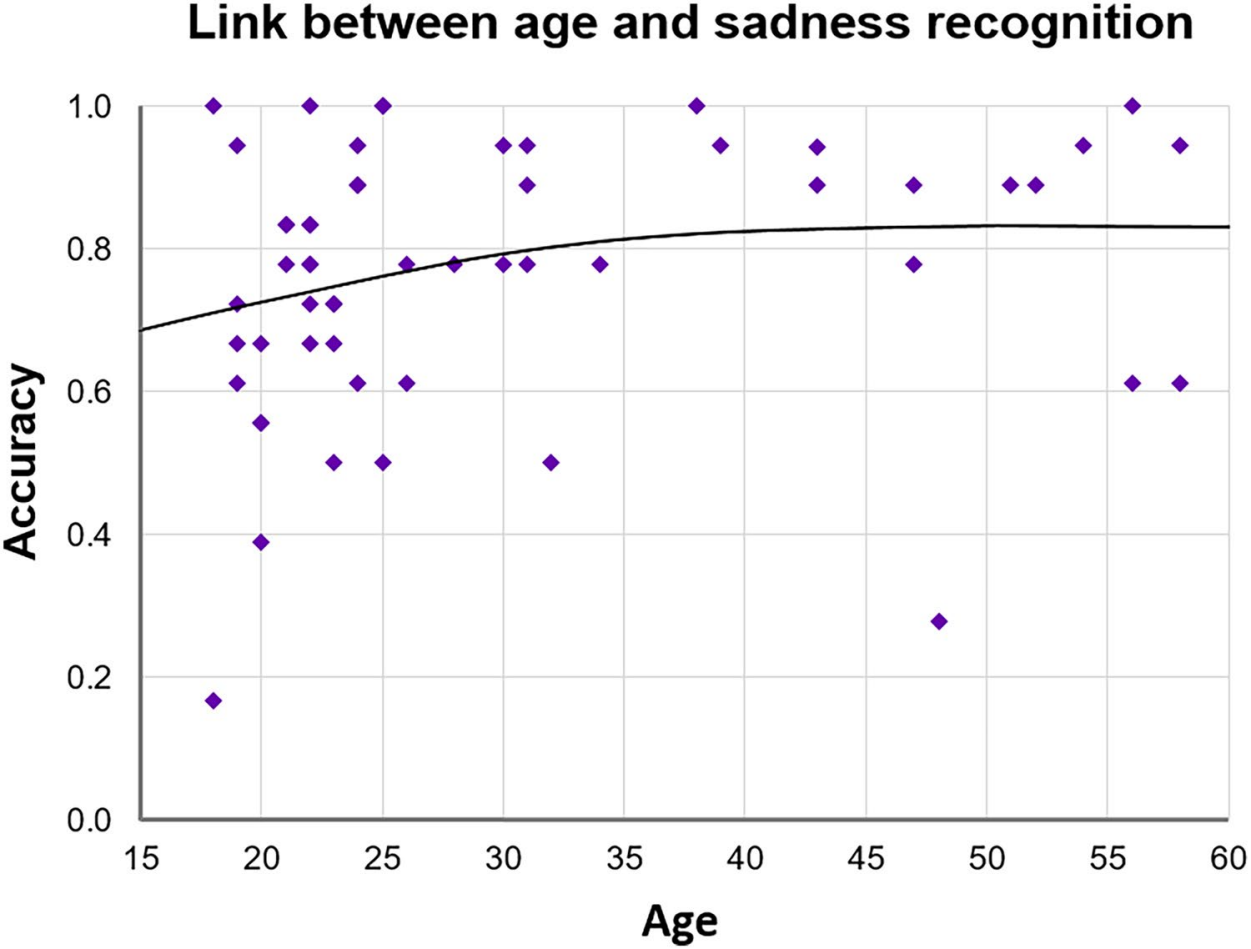
Link between inferring sadness on the EMF task and age. Significant positive non-linear Spearman correlation (*p* = 0.039) was found between recognition accuracy of sadness and age.

Although the overall recognition rate tended to positively correlate with chronological age (Spearman’s rho, *ρ*(53) = 0.261, *p* = 0.054), only sadness showed a significant non-linear positive link with age (*ρ*(53) = 0.280, *p* = 0.039). As seen in Fig. 3, younger women exhibited rather high variability in inferring sadness. Interestingly, recognition of sadness and disgust was non-linearly correlated with each other in our sample of female TD participants (*ρ*(53) = 0.308, *p* = 0.022).

### Recognition accuracy for male and female posers

Individual correct response rates were submitted to a two-way repeated measures ANOVA with the within-subject factors Emotion (angry, happy, neutral, sad, fearful, disgusted) and Gender of Poser (female/male). [Information about the gender of posers was corrupted in the data sets of two participants.] A main effect of Gender of Poser was not significant (*F*(1,516) = 0.94, *p* = 0.333, n.s.). A main effect of Emotion was highly significant (*F*(5,516) = 80.92, *p* < 0.001, effect size, *η*^2^ = 0.61) with a significant Gender by Emotion interaction (*F*(5,516) = 14.31, *p* < 0.001, *η*^2^ = 0.21).

**Figure 4:**
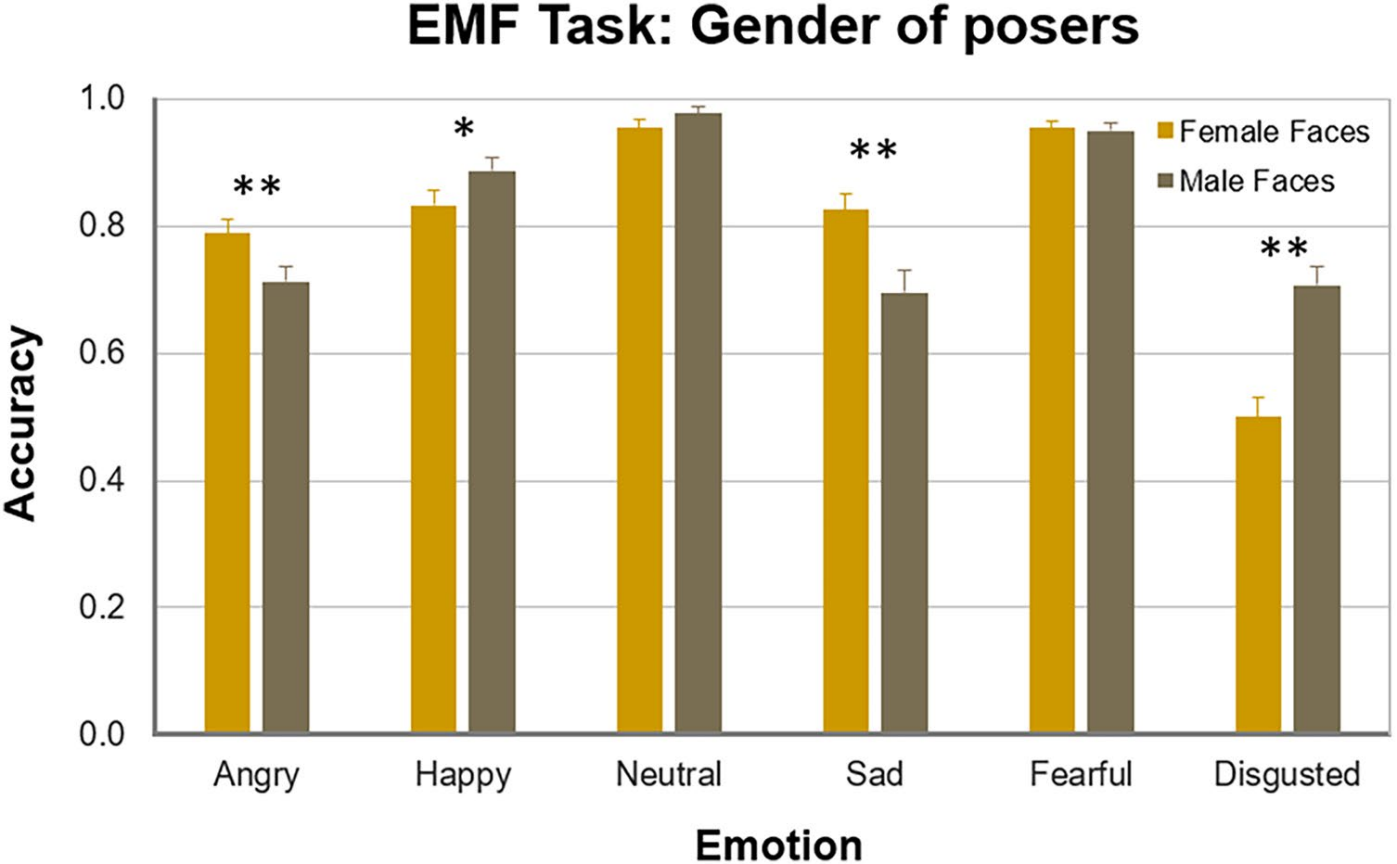
Recognition accuracy of facial emotions on the EMF task for female faces (yellow mustard) and male (olive grey) faces in TD females. Vertical bars represent ± SEM. Double asterisks indicate significant differences (*p* < 0.05), single asterisk a tendency.

As seen in Fig. 4, inferring sadness in the eyes of females was more accurate compared to male posers (Wilcoxon signed-rank test, *z* = 3.45, *p* = 0.003; here and further two-tailed and FDR corrected for multiple comparisons; Cohen’s *d* = 1.08). Inferring anger (*z* = 2.48, *p* = 0.028, *d* = 0.72) was also better in female eyes. By contrast, disgust was, and happiness tended to be, better recognizable in the eyes of males (disgust, *z* = 4.40, *p* < 0.001, *d* = 1.52; happiness, *z* = 1.95, *p* = 0.077). Inferring neutral expressions and fear did not depend on the gender of the poser (Table S2, Supplementary Material).

For female posers, sadness recognition tended to positively correlate (*ρ*(51) = 0.243; *p* = 0.077), whereas disgust recognition positively correlated (*ρ*(51) = 0.355, *p* = 0.008) with beholder age. For male posers, recognition of fear (*ρ*(51) = 0.326, *p* = 0.016) and anger (*ρ*(51) = 0.278; *p* = 0.041) positively correlated with age.

## Discussion

The main outcome of the study indicates that when visual input from the eyes only is available, in healthy women, recognition of such a subtle emotional expression as sadness substantially improves across the lifespan, from young to mid-adulthood. It appears that improvement in inferring sadness in female eyes primarily contributes to this result, as sadness recognition in the eyes of male posers does not correlate with age. At first glance, it may appear paradoxical, as masks have a more pronounced impact on inferring sadness in the eyes of males compared to females.

Several factors may potentially contribute to this outcome. First of all, although both the upper (the eyes and surrounding part of a face) and lower parts of a face are believed to essentially contribute to sadness recognition^36,37^, its recognition accuracy as well as perceived intensity and confidence in recognition are severely affected by masks^1–16,38^. This leads to the conclusion that not only visual information revealed in the eyes is necessary for efficient sadness recognition. This outcome appears startling, as the eyes are traditionally believed to be particularly vital for sadness recognition*.* In the Facial Action Coding System (FACS^39^), most facial action units (AUs) engaged in sadness expression come from the eye region: AU1, inner eyebrow raiser; AU4, brow furrower; AU43, upper eyelid lower; AU64, eyes down, and only AU15, lip corner depressor is beyond the eye region^40^.

Second, expression of emotions in the eyes is/may be gender-dependent. Social norms often encourage women to be more emotionally expressive, while men are typically socialized to exhibit less emotional variability^41^. This societal influence likely contributes to differences in recognition accuracy, as women, for example, are more prone to use their cheek-raisers while smiling than men, resulting in a smile that appears more genuine (also known as a true or Duchenne smile^42^). This nicely dovetails with the present findings as far as poorer recognition of sadness (and anger) in male posers may be explained by a lower intensity of sadness in the male eyes. By contrast, however, disgust and happiness tend to be better recognizable in male eyes, which questions the greater poignancy of females in all emotional expressions.

In addition, as pointed out earlier^17^, in face images routinely used in experimental settings and face databases, facial emotions are displayed by professionals who have been (i) asked and (ii) trained to express emotions. These expressions may turn out to be rather different from the natural feelings we experience and express in real life. In naturalistic environments, we are usually quite far from extreme affect demonstrations.

The ability to express (and read) emotions in the eyes had been plentifully reflected in poetry, for instance, for the eyes expressing hatred, ‘*As if there were four, though they are three, stare at him hungrily*’ (Alexander S. Pushkin, The Tale of Tsar Saltan, 1831; translated by Marina A. Pavlova).

**Figure 5:**
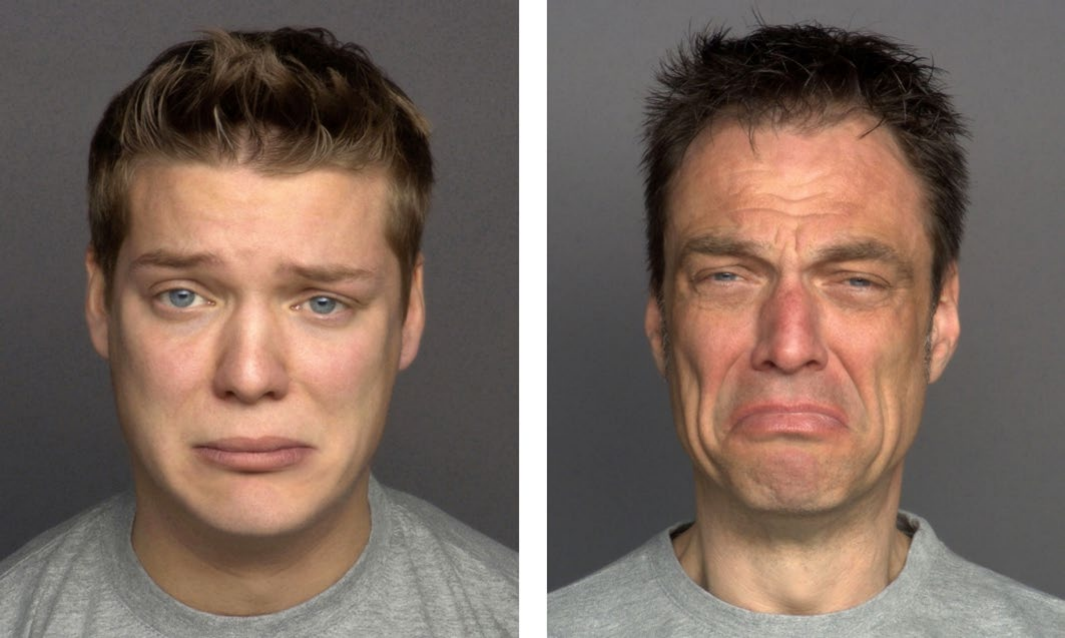
Pronounced differences in expression of sadness within the same cultural background. The images are taken from the MPI FACES database (Ebner et al.^35^; public domain). These images are presented for illustrative purposes only, and have not been used as experimental material in the present study.

Third, cultural/ethnical background in emotion expression and experience may contribute to the efficiency of emotion recognition in the eyes as well^18^. For instance, face masks hamper recognition of happiness in U.S. Americans but not in Japanese individuals, suggesting a higher sensitivity of Japanese people to information available in the eyes^34^. Individualism is reported to be associated with better recognition of (unmasked) happiness but poorer recognition of (unmasked) sadness^43^. On the other hand, in a U.S. sample, Asian-born participants exhibit the lowest sadness recognition accuracy compared to both Asian Americans and European Americans^44^. In light of these findings, the present outcome has to be taken with caution, as both expression of sadness through the eyes (faces were taken from the Max Planck Institute FACES database, German posers^35^) as well as inferring of sadness in the eyes by German beholders may reflect specific cultural particularities. As seen in Fig. 5, however, even within the same culture, individual differences in expression of sadness (also by the eyes) may be rather pronounced.

In a nutshell, the present findings suggest that in inferring subtle emotional expressions (such as sadness) in the eyes, experience obtained with age, from young through middle healthy adulthood, may be of substantial benefit. At the same time, sadness recognition may be modulated by estrogen receptor gene polymorphisms^45^. In young women, recognition accuracy of sadness (and disgust) is higher in the follicular phase (with lower levels of estrogen and progesterone) than in the menstrual phase^46,47^. Overall, females are recently reported to be faster than males in sadness recognition; moreover, both female and male observers are more accurate in inferring sadness in female as compared with male faces^48^. A better understanding of possible alterations in reading emotions in the eyes with age calls for future tailored work in male observers.

### Supplementary Information

Supplementary Tables.

## Data Availability

Data is provided within the manuscript or supplementary information files.
