# Supplementary material for: ‘The mirror of the soul?’ Inferring sadness in the eyes
Source: Sci Rep. 2024 Aug 29;14:20063. doi: 10.1038/s41598-024-68178-0 (PMC11362606; doi:10.1038/s41598-024-68178-0)
Supplement: Supplementary file 1 — Supplementary Tables. [file 41598_2024_68178_MOESM1_ESM.pdf]

‘The mirror of the soul?’ Inferring sadness in the eyes

Jonas Moosavi[1], Annika Resch[1], Alexander N. Sokolov[1], Andreas J. Fallgatter[1,2], Marina A. Pavlova[1]

[1] Social Neuroscience Unit, Department of Psychiatry and Psychotherapy, Tübingen Center for Mental Health (TüCMH), Medical School and University Hospital, Eberhard Karls University of Tübingen, Tübingen, Germany

[2] German Center for Mental Health (DZPG), Partner Site Tübingen, Germany

Supplementary Material

Table S1. Recognition accuracy: group comparison

	Group 1			Group 2					
	**Mean**	**Mdn**	**95% CI**	**Mean**	**Mdn**	**95% CI**	**Mann-Whitney *U* / *t* - test**	***P* uncorr.**	***P* corr.**
**anger**	0.71 ± 0.15			0.78 ± 0.10	0.83	[0.75; 0.82]	*U* = 257	0.047**	0.094*
**happiness**	0.84 ± 0.14	0.89	[0.79; 0.89]	0.88 ± 0.13	0.89	[0.83; 0.93]	*U* = 282	0.165	0.198
**neutral**	0.96 ± 0.08	1.00	[0.93; 0.99]	0.97 ± 0.08	1.00	[0.94; 1.00]	*U* = 340	0.555	0.555
**sadness**	0.69 ± 0.19			0.83 ± 0.16	0.89	[0.77; 0.89]	*U* = 201	0.003**	0.018**
**fear**	0.94 ± 0.07	0.94	[0.91; 0.96]	0.97 ± 0.05	1.00	[0.95; 0.99]	*U* = 269	0.075*	0.112
**disgust**	0.56 ± 0.17			0.65 ± 0.16			*t*(53) = 2.09	0.041**	0.094*

Table S2. Recognition accuracy for male and female posers

	female faces			male faces				
	**Mean**	**Mdn**	**95% CI**	**Mean**	**Mdn**	**95% CI**	**Wilcoxon signed-rank test, *z***	***P* corr.**
**anger**	0.79 ± 0.16	0.78	[0.75; 0.83]	0.71 ± 0.17	0.78	[0.67; 0.76]	2.48	0.028**
**happiness**	0.83 ± 0.18	0.89	[0.78; 0.88]	0.89 ± 0.16	0.89	[0.84; 0.93]	1.95	0.077*
**neutral**	0.95 ± 0.10	1.00	[0.93; 0.98]	0.98 ± 0.07	1.00	[0.96; 1.00]	1.54	0.149
**sadness**	0.83 ± 0.18	0.89	[0.78; 0.88]	0.70 ± 0.26	0.78	[0.62; 0.77]	3.45	0.003**
**fear**	0.95 ± 0.08	1.00	[0.93; 0.98]	0.95 ± 0.09	1.00	[0.93; 0.98]	0.23	0.818
**disgust**	0.50 ± 0.22			0.71 ± 0.23	0.78	[0.64; 0.77]	4.40	< 0.001**

Note: For non-normally distributed data, additionally to means and SDs, Mdns and 95% CIs are reported.
Double asterisks indicate significant differences ($p < 0.05$), single asterisks indicate a tendency ($0.05 < p < 0.1$).
